# Size and Methylation Index of Cell-Free and Cell-Surface-Bound DNA in Blood of Breast Cancer Patients in the Contest of Liquid Biopsy

**DOI:** 10.3390/ijms23168919

**Published:** 2022-08-10

**Authors:** Svetlana Tamkovich, Alexey Tupikin, Anton Kozyakov, Pavel Laktionov

**Affiliations:** 1 Institute of Chemical Biology and Fundamental Medicine, Siberian Branch of Russian Academy of Sciences, Novosibirsk 630090, Russia; 2 Department of Clinical Biochemistry, V. Zelman Institute for Medicine and Psychology, Novosibirsk State University, Novosibirsk 630090, Russia; 3 Department of Mammology, Novosibirsk Regional Clinical Oncological Dispensary, Novosibirsk 630108, Russia

**Keywords:** circulating DNA, cell-free DNA, cell surface bound DNA, fragment sizes, methylation, breast cancer, liquid biopsy

## Abstract

Aberrantly methylated circulating DNA (cirDNA) has proven to be a good cancer marker, but its detection is limited by low concentrations, fragmentation, and insufficiency. Since the methylated cirDNA was shown to be more stable in circulation than the unmethylated one and was shown to bind with the blood cell surface, we studied the concentration, representation, and fragmentation of tumor-derived methylated DNA in cell-free and cell-surface-associated DNA. We found that long DNA fragments (more than 10 kb) are mainly associated with the surface of blood cells. However, in plasma short DNA fragments (100–1000 bp) were also found along with long DNA fragments. Isolation of short fragments after separation of cirDNA in 6% PAGE followed by quantitative PCR (L1 element) has shown that short DNA fragments in healthy females represent 22% versus 0.5–4.4% in breast cancer patients. The methylated form of the *RARβ2* gene was detected only in long DNA fragments by Real-time TaqMan PCR of bisulfite-converted DNA. The methylation index of cirDNA from healthy women was estimated at 0%, 9%, and 7% in plasma, PBS-EDTA, and trypsin eluates from the surface of blood cells, respectively. The methylation index of breast cancer patients’ DNA was found to be 33%, 15%, and 61% in the same fractions confirming the overrepresentation of methylated DNA in csbDNA.

## 1. Introduction

Cancer-specific DNA with genetic and epigenetic alterations identical to tumor DNA usually circulates in the blood of patients with oncological diseases [1]. Epigenetic DNA markers, namely aberrantly methylated DNA, are on the list of potential circulating tumor markers since the aberrant methylation of oncogenes and tumor suppressor genes is known to be one of the earliest events during tumor cell transformation, and it is efficiently detected in the abundance of normal, non-methylated DNA and apt to longer circulation in blood [2,3]. Recently two non-invasive diagnostic tests based on tumor-specific sequences in circulating cell-free DNA (cfDNA) using real-time quantitative PCR, have been approved for clinical use. In particular, the analysis of the methylation of the genes *Septin 9* and *SHOX2* in blood plasma cirDNA allows for detecting colorectal and lung cancer with sensitivity and specificity greater than 90% [4,5]. Both the Epi proColon^®^ and Epi proLung^®^ tests have been approved for diagnostic use in China and the European Union, with the former test being also approved by the FDA in the United States (http://www.epigenomics.com, accessed on 11 December 2021).

Unfortunately, liquid biopsy tests based on aberrantly methylated DNA analysis are not so efficient for other tumors. The low sensitivity (the most significant criteria for practical use) of non-invasive diagnostic tests is affected by the low concentration and fragmentation of cfDNA in the bloodstream aggravated by additional fragmentation caused by bisulfite treatment [6,7].

Tumor specific cfDNA was found in short DNA fragments generated by active secretion or the apoptosis of cancer cells, and in longer fragments originating in necrosis and autophagy [8,9,10,11,12]. Due to its origin, cfDNA is fragmented and circulates in the bloodstream as nucleoprotein complexes [7], and it is additionally degraded by nucleases and proteases in the blood [13,14]. The total hydrolase activity in the blood depends on the concentrations of proenzymes/enzymes and activators/inhibitors [15,16], and may be related to tumor histotype/carcinogenicity.

The inconsistency of literature data on the size of DNA fragments and ways of cfDNA fragmentation [17] complicates the design of PCR systems, which, in turn, can lead to significantly different efficiencies in detection of the same epigenetic markers in plasma samples from cancer patients with a similar clinical manifestation [18]. The potential diagnostic value of non-invasive tests can be strengthened by the use of cell-surface-bound DNA (csbDNA), namely blood cell surface-associated circulating DNA. For example, the methylation index of csbDNA from the blood of NSCLC patients was found to be more diagnostically efficient than cfDNA [19].

To confirm the hypothesis of a greater representativeness of epigenetic markers in csbDNA compared to cfDNA, breast cancer was chosen as one of the most represented nosologies [20], and aberrantly methylated *RARβ2* was chosen as one of the most studied tumor biomarkers [21]. Moreover, *RARβ2* was selected in the present study since hypermethylation frequency for this gene was reported to be significantly higher in breast tumors compared with normal breast tissues and due to it being detected in cfDNA and csbDNA [22,23,24].

Here we present the data of a complex study on different cirDNA fractions in blood of breast cancer patients (BCPs) and healthy females (HFs), namely cell-free and cell-surface-bound DNA, in respect to their fragmentation and representation of methylated DNA, in order to evaluate the usefulness of these DNA pools as a source of aberrantly methylated DNA for breast cancer diagnostics.

## 2. Results

### 2.1. Concentration and Size of cf DNA and csbDNA Fragments in Blood of HFs and Untreated BCPs

**Figure 1:**
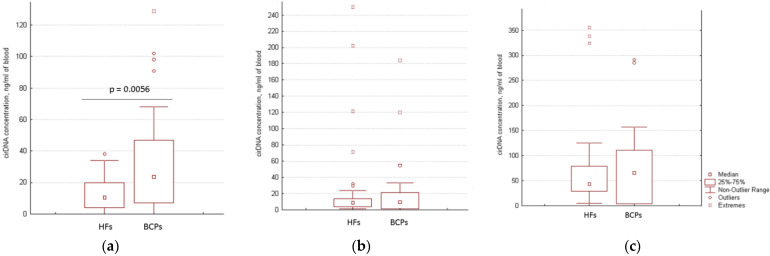
DNA concentration of different blood-circulating DNA fractions. (**a**) Plasma cfDNA; (**b**) csbDNA in PBS–EDTA eluate; (**c**) csbDNA in trypsin eluate. The data are recalculated to initial blood volume.

The cirDNA concentration in the plasma and at the surface of blood cells of HFs (*n* = 50) and BCPs (*n* = 26) was estimated by qPCR specific for LINE-1. A significant increase of the plasma cirDNA concentration was found for BCPs compared with HFs (median 23.5 versus 10.5 ng/mL of total blood, *p* = 0.0056, Mann-Whitney U test) (Figure 1a). The revealed difference between the tumor patients and the controls coincide with published data [25,26]. At the same time, no significant differences were found for csbDNA in BCPs and HFs (in the PBS-EDTA fraction median of csbDNA was 8.5 versus 10.3 ng/mL of total blood (Figure 1b), and in the trypsin fraction the median of csbDNA was 66 versus 43 ng/mL of total blood (Figure 1c), respectively.

**Figure 2:**
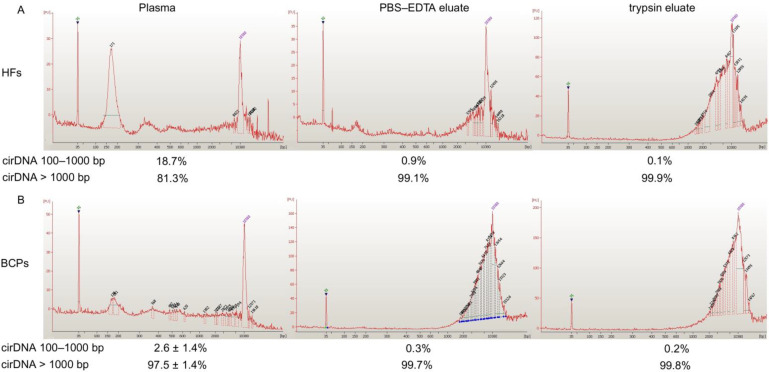
Size distribution of DNA circulating in the blood of HFs (**A**) and BCPs (**B**). Data of Agilent 2100 Bioanalyser^TM^ assay. Typical DNA size distribution in blood of BCP is presented.

Plasma DNA and csbDNA were characterized by an Agilent 2100 Bioanalyser^TM^ using a High Sensitivity DNA Kit and short DNA fragments isolated by electroelution after 6% PAAG electrophoresis. It was found that bulk DNA from normal plasma mainly contains DNA fragments ~171–180 bp and fragments of 8 and 13.81 kbp to a much smaller extent. Only long DNA fragments were found in csbDNA from HFs (from 1.52 to 14.49 kbp) (Figure 2A). All BCP cfDNA samples were analyzed individually and mainly contained long DNA fragments and fragments of ~171 and ~180 bp to a much smaller extent. csbDNA from BCP had a similar size distribution profile as that in HFs (Figure 2B).

The efficacies of 161 b.p. PCR product isolation by electroelution after 6% PAAG electrophoresis were 91% and 80% for 1 ng and 40 pg of DNA, respectively. The ratio of DNA from 100 bp to 1 kbp to total cirDNA in plasma and csbDNA was quantified by Q-PCR for LINE1 repeats. It was found that less than 20 % of HF plasma DNA were represented by short DNA (100–1000 bp), whereas only ~2% of DNA isolated from BCP blood plasma had a similar size range (Figure 2). Short DNA is rarely present in csbDNA (0.1–0.9 %). The data are close to those reported previously [27,28,29], although they differ from the data of other studies [30].

### 2.2. Distribution of Aberrantly Methylated and Unmethylated RARβ2 in Short DNA and Total DNA from Blood of HFs and BCPs

Our earlier studies have shown an increase in the methylated gene detection sensitivity when csbDNA was used alone or in combination with cfDNA [19,22]. In the current study, we measured the index of gene methylation (IM) in fractions of cfDNA and csbDNA by the Q-PCR of RARβ2. It was found that aberrantly methylated RARβ2 is detected only in long DNA (Table 1). Moreover, while also found in HFs methylated RARβ2 DNA IM did not exceed 10%, whereas in BCPs blood total DNA IM overexceed 60% (Table 1).

These data demonstrate that the most valuable source of tumor aberrantly methylated DNA for “liquid” biopsy is csbDNA. Firstly, the fragmentation of csbDNA in BCP blood prevents the influence of natural DNA fragmentation on the PCR assay data [17].

Secondly, csbDNA is enriched in methylated DNA as confirmed by IM of RARβ2. Thus, despite some inconvenience in csbDNA isolation, this fraction of DNA demonstrates obvious benefits as a source of tumor specific methylated DNA.

### 2.3. Concentration of Aberrantly Methylated RARβ2 in Blood of Untreated Luminal BCPs at I-II Stages of Disease

**Figure 3:**
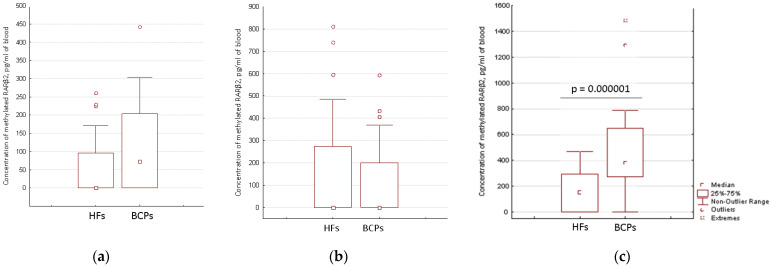
Concentration of aberrantly methylated RARβ2 in blood of HFs and BCPs. (**a**) Plasma; (**b**) PBS–EDTA eluate; (**c**) Trypsin eluate.

The aberrantly methylated RARβ2 concentration in cfDNA and csbDNA samples from the blood of HFs (*n* = 46) and untreated BCPs (*n* = 23) at T1-T2 stages was measured by MSP. The median concentration of the aberrantly methylated RARβ2 gene in plasma of BCPs was found to be about 72 pg/mL of blood (DNA was detected in 52% (12/23) samples with range 0–442 pg/mL). In plasma of HFs, methylated RARβ2 was detected in 37% (17/46) of samples with a range 0–261 pg/mL (Figure 3a). There were no significant differences found between the methylated csbDNA concentrations in healthy and cancer states in PBS-EDTA eluates (Figure 3b), with only 35% and 26% of samples containing methylated RARβ2. A significant increase in the trypsin eluates methylated csbDNA concentrations was found in BCPs as compared to HFs (median 381 versus 151 pg/mL of blood, Mann-Whitney U test) (Figure 3c). Moreover, the aberrantly methylated RARβ2 gene was found in 96% (22/23) of BCPs samples with a range of 0–1484 pg/mL and 57% (26/43) of HFs samples with a range of 0–470 pg/mL. The revealed difference between the cancer patients and the controls coincide with published data for lung cancer [19].

**Figure 4:**
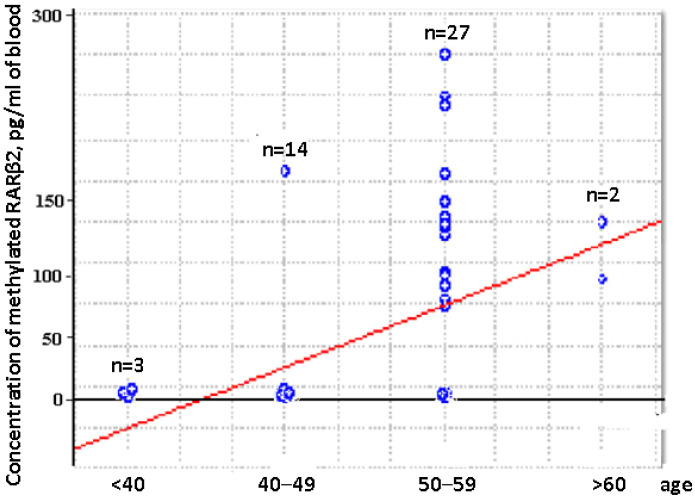
Dependence of the aberrantly methylated RARβ2 gene plasma concentration and the age of HFs.

Comparative analysis of the aberrantly methylated RARβ2 gene concentration in cfDNA and csbDNA of BCPs revealed the significantly increased concentration of tumor-associated DNA in trypsin eluates in comparison to plasma and PBS-EDTA eluates (381 vs. 72 pg/mL, *p* = 0.000001 and 381 vs. 0 pg/mL, *p* = 0.00001, respectively). This data coincide with the data on RARβ2 gene IM in the blood of BCPs (Table 1). There was no association found between the aberrantly methylated RARβ2 gene concentration values in plasma DNA and csbDNA with tumor stages or regional lymph node metastasis. Nevertheless, a significant correlation was found between increased plasma methylated DNA concentration and the age of HFs (Spearman coefficient R = 0.45, *p* = 0.001) (Figure 4).

Thus, the formation of an age-comparable comparison group is extremely important in assessing the diagnostic significance of methylated cirDNA markers.

Distribution data of methylated DNA (Figure 3) led to the decision to combine two fractions of csbDNA (csbDNA from the PBS eluate and csbDNA from the trypsin eluate). A comparison using the receiver operating characteristic (ROC) curve analysis of the diagnostic significance of cfDNA and combined csbDNA revealed the higher diagnostic significance for csbDNA (Table 2).

It should be noted that the usage of total cirDNA (cfDNA and csbDNA) slightly worsened the efficiency of differentiation between HFs and luminal BCPs at I and II stages of the disease.

## 3. Discussion

Currently, circulating tumor cells, cfDNA, small RNAs, and exosomes are used for liquid biopsy [31,32,33]. The advantages of circulating genetic markers include the opportunity to detect neoplasia-associated point mutations, deletions/inserts, translocations, and amplifications, as well as aberrant cytosine methylation in the CpG-dinucleotides of tumor suppressor genes [1]. To date, aberrant DNA methylation has been shown to be one of the most common and early causes of malignant cell transformation and tumors of different localizations, including breast cancer. Moreover, these circulating epigenetic tumor markers were shown to have analytical benefits, related to chemical conversion, as compared to point mutation, copy number variation or DNA rearrangements [34]. Indeed, many studies have reported the hypermethylation of various tumor suppressor genes in breast tumors, including the *RARβ2* gene, which is shown to be methylated at a high frequency [35]. *RARβ2*, the gene encoding the retinoid acid nuclear receptor, plays a critical role during embryonic development, homeostasis, cell growth and differentiation. *RARβ2* has been shown to be methylated in 10.4% of cancer tissue compared to 0% in adjacent normal breast tissue [24]. According to other data, the percentage of *RARβ2* aberrant promoter methylation in tumor tissue is higher −46% versus 4% in breast normal tissue [36]. Despite the fact that the increase in *RARβ2* methylation was lower in plasma cirDNA samples compared to tumor tissues [37,38], this epigenetic cancer marker is considered promising for the use in diagnostic panels being developed for liquid biopsy. Moreover, in our early studies we observed the increase of its accuracy in csbDNA [19,22].

It should be noted that cancer-related DNA usually represents a minor part of cfDNA and is found in low concentrations, thus requiring high PCR specificity [39,40]. To increase the amount of analyzed tumor DNA, either an increase in venous blood volume is required (which is extremely problematic for patients with advanced stages accompanied by anemia, while there is no increase in the ratio of methylated and unmethylated forms) or the use of more abundant blood-circulating source of extracellular DNA like csbDNA is required. The concentration of cfDNA and their size/fragmentation are determined not only by uneven fragmentation of the genome during cell death, but also by nucleases and proteases that hydrolyze cfDNA hidden in nucleoprotein complexes [13,14,17]. These cfDNA features impose various restrictions on both isolation methods and subsequent analysis [3]. According to some data, the cfDNA of cancer patients has a high fragmentation, while according to others, short and long fragments are equally represented [8]. Thus, there is no consensus in the literature about the integrity index of cfDNA, which is extremely important for the development of PCR systems for liquid biopsy. Since methylated DNA has been shown to be more stable than unmethylated [41] and to easily bind with the cell surface where it is less accessible to hydrolases, it should be assumed that csbDNA has a higher molecular weight than plasma DNA and a higher diagnostic value. In the current study, the presence of high molecular weight DNA on the blood cell surface was confirmed. Moreover, trypsin eluate csbDNA from cancer patients′ blood has had an increased IM compared to plasma DNA. Earlier studies have shown that the sensitivity of the methylated gene detection is increased when cfDNA and csbDNA from the gastric and lung cancer patients blood plasma are analyzed simultaneously [19,42]. These differences can be associated both with different neologies and with different designs of the PCR systems used. However, the authors have also shown that cfDNA methylation analysis alone is not sufficient for successful cancer detection. The reasons for the decrease in the effectiveness of PCR may be the methylation mode, when not all CpG dinucleotides in the promoter region are simultaneously methylated [37], as well as allele-specific methylation [43]. At the same time, some new approaches, such as the use of NGS sequencing targeting a specific locus make it possible to overcome such limitations [43]. It should be mentioned that the low level of aberrantly methylated DNA in cfDNA represents a serious limitation in all methods aimed at analyzing tumor DNA circulating in the blood.

The dependence of the aberrantly methylated *RARβ2* gene concentration in blood plasma with HF age, revealed in this study, indicates the extreme importance of an age-comparable control group for the assessment of the methylated DNA markers′ diagnostic significance. To date, various causes of age-related changes in methylation of tumor suppressor genes have been identified. In particular, an increase in the degree of circulating DNA methylation is associated with a change in the activity of the methylation system as well as with the influence of carcinogens and environmental factors [42,44,45].

The reasons why tumor DNA is bound to the surface of blood cells are not clear, but this relationship may be related to the structure of nucleoprotein complexes, as well as cancer-induced changes in the composition and the amount of the blood cell-surface proteins and blood plasma proteins [26,46]. It has been shown that during apoptosis some DNA sequences are more abundant in the cfDNA pool than others [47].

In summary, we would like to emphasize that high molecular weight highly methylated DNA bound to the surface of blood cells is a promising source of DNA molecules for cancer diagnosis. Undoubtedly, the further search for tumor markers is necessary to improve diagnostic system efficacy. Furthermore, these molecules could be used in a multi-marker approach currently used to increase the sensitivity and specificity of non-invasive liquid biopsy tests.

## 4. Materials and Methods

### 4.1. Patients and Blood Treatment 

Blood samples from HFs (*n* = 50, median age 52) were obtained from Novosibirsk Central Clinical Hospital. HFs did not have any female disorders (dysplasia, endometriosis, etc.) or any malignant diseases.

Blood samples from untreated BCPs (*n* = 26, median age 57) were obtained from Novosibirsk Regional Oncology Dispensary. The clinicopathological parameters of the patients with breast cancer are presented in Table 3.

The subtype of breast cancer was established by the immunohistochemical study of tissue samples after surgery (expression of receptors for estrogen (ER) and progesterone (PR)), HER-2 status and the level of proliferative activity (expression of Ki67) in accordance with the St. Gallen Consensus Recommendation [48]. IHC was prepared as described [49]. For ER and PR expressions, the cases were classified as positive when nuclear immunoreactivity was in ≥1% of tumor cells according to the American Society of Clinical Oncology/College of American Pathologists (ASCO/CAP) guidelines [50]. Sections stained with ER and PR were scored using the H-score method [51]. HER2 protein-positive status was defined as a score of 3+ by IHC or 2+ by IHC together with the confirmed *c-erbB2* gene amplification by fluorescence in situ hybridization (FISH).

Venous blood (9 mL) was collected in K_3_EDTA spray-coated vacutainers (Improvacuter, China, cat. no. 694091210), immediately mixed using a rotary mixer, placed at +4 °C, and fractionated into plasma and blood cells within an hour after blood sampling. Blood was centrifuged at 290× *g* for 20 min. Blood plasma was then transferred into a new tube and centrifuged a second time at 1200× *g* for 20 min. Csb-DNA was eluted from the blood cell surface with PBS supplied with 5 mM EDTA (PBS-EDTA) and trypsin solutions, as previously described [52]. Briefly, cells were washed with nine volumes of PBS-EDTA and centrifuged as plasma (290× *g* for 20 min, after that 1200× *g* for 20 min), and the supernatant was collected as PBS-EDTA eluate. Next, an equal volume of 0.25% trypsin (Sigma, T-4799 (Louis, MO, USA)) in PBS-EDTA was added to the pelleted cells and incubated using a rotary mixer for vacutainers (10 rpm) for 4.5 min at room temperature, with subsequent inactivation of the enzyme by the addition of 1/10 sample volume of 10× trypsin inhibitor solution (Sigma, T-9003, 3.2 mg/mL (Louis, MO, USA)). Cells were then pelleted by centrifugation as described above, and the supernatant was collected as trypsin eluate. Plasma, PBS-EDTA and trypsin eluates were stored at −80 °C in aliquots and defrosted before DNA isolation.

Before subsequent manipulations, all samples were tested for the absence of hemolysis/lysis of blood cells by assessing the level of hemoglobin (the absorbance of <0.175 at 414 nm). Blood samples with signs of hemolysis that occurred in cancer patients due to the general disturbance of lipid metabolism were excluded from the study.

### 4.2. CirDNA Isolation and Quantification of Short Fragments in Cell-Free and csb-DNA

CirDNA was isolated from 3 mL of plasma samples, 20 mL of PBS-EDTA samples and 5 mL of trypsin samples using the “DNA Isolation Kit” (BioSilica Ltd., Novosibirsk, Russia) according to the manufacturer′s protocols and concentrated by precipitation in acetone as triethylammonium salts [53].

The concentration of isolated DNA was measured by quantitative polymerase chain reaction (Q-PCR) specific for long interspersed nuclear element 1 (LINE-1) repetitive elements [53]. The Q-PCR was performed with an ICycler iQ5 (Bio-Rad, Hercules, CA, USA) in a total reaction volume of 30 μL containing 5 μL of DNA; 600 nM of each primer (Table 2); 300 nM probe (Table 2); 2.5 mM deoxynucleotide triphosphates and Taq polymerase buffer (containing 65 mM tris-HCl, 16 mM (NH_4_)_2_SO_4_, 0.05% Tween-20, 6 mM MgCl_2_, pH 8.8) and 1 U of Taq polymerase (Biolabmix, Novosibirsk, Russia). PCR was performed under the following conditions: denaturation at 95 °C for 4.5 min, followed by 45 cycles at 95 °C for 15 s and 60 °C for 45 s. Genomic DNA from human leukocytes served as a standard for obtaining the calibration curves. The DNA concentration was estimated according to the initial volume of each blood sample.

To study DNA fragmentation, half of cirDNA from normal blood samples (n = 25) were mixed to generate one sample from HFs, and DNA from all BCP blood samples (n = 8) were analyzed individually. The size of cirDNA in plasma and in eluates from the blood cell surface was evaluated using an “Agilent High Sensitivity DNA Kit” and an Agilent 2100 Bioanalyser ^TM^ (Agilent Technologies, Waldbronn, Germany).

To estimate the share of short fragments in DNA isolated from plasma, PBS-EDTA and trypsin eluate DNA samples were separated by 6 % PAAG, and short fragments (100–1000 bp) were isolated by electro-elution at 200 V for 2 h. DNA was eluted from DEAE-cellulose with washing by five times of 20 µL of 3 M LiClO_4_ in water and precipitated with 1 mL of 2% lithium perchlorate solution in acetone. The precipitate was collected by centrifugation at 13,000 rpm for 10 min, DNA was washed from the rest of the salts with acetone, dried at room temperature, and dissolved in 30 μL of water. Short isolated cirDNA were quantified by TaqMan PCR for LINE-1 repeats as described above. The efficacy of short DNA isolation was confirmed by isolation from the gel of 1 ng and 40 pg of PCR product of *RARβ2* (GenBank X56849.1, 924-1117, 194 bp).

### 4.3. Bisulfite Conversion and Methyl-Specific TaqMan PCR (MSP)

Bisulfite treatment was performed using a EZ DNA Methylation-Gold ™ Kit (ZymoResearch, Irvine, CA, USA) according to the manufacturer’s instructions. Isolated total DNA and short DNA fragments from plasma, from PBS-EDTA eluates and from trypsin eluates or genomic DNA or PCR products of *RARβ2* (independent of methylation and methylspecific, GenBank X56849.1 931-1116, 186 b.p. and GenBank X56849.1 926-1116, 161 b.p., respectively) were treated simultaneously. Bisulfite-treated cirDNA was eluted from DNA spin columns in 40 μL of an elution buffer and stored in aliquots at −40 °C.

Concentrations of methylated and independent to methylation forms of the *RARβ2* gene were assessed by quantitative MSP. The MSP was performed with an ICycler iQ5 (Bio-Rad, Hercules, CA, USA) in a total reaction volume of 30 μL containing 5 μL of DNA; 300 nM of each primer (Table 4); 0.5 mM deoxynucleotide triphosphates, Taq polymerase buffer (containing 65 mM tris-HCl, 16 mM (NH_4_)_2_SO_4_, 3.5 mM MgCl_2_, 0.05% Tween-20, 1× SYBR Green I, 10 mM fluorescein, pH 8.8) and 1 U of Taq polymerase. A PCR was performed under the following conditions: denaturation at 95 °C for 3 min, followed by 40 cycles at 95 °C for 30 s, 60 °C for 5 s and 72 °C for 30 s. Genomic DNA from human leukocytes and PCR products served as a standard for obtaining the calibration curves. The DNA concentration was estimated according to the initial volume of each blood sample.

The coefficient of variation between repeats of measurements for *RARβ2* was 10%; the sensitivity of five gene copies/μL of DNA and the MSP efficiency was in the range of 93–95%. Standard curves were generated using serial dilutions of the purified methylated and unmethylated MSP amplification products, stored frozen as stock solutions (10^12^ copies/mL) and freshly diluted before each use. The index of gene methylation (IM) was calculated as % IM = 100 × [copy numbers of methylated *RARβ2*/(copy numbers of methylated *RARβ2* + unmethylated *RARβ2*)].

### 4.4. Data Analysis

A statistical analysis was performed using the Statistica 6.0 software and the GraphPad PRISM 5 software (GraphPad Software, La Jolla, CA, USA). The assessment of the normal distribution of the results was performed using the Kolmogorov-Smirnov test. The significance of differences was assessed using the nonparametric Mann–Whitney test. Data are presented as the median and the upper and lower quartiles. For the *RARβ2* gene, samples were categorized as methylated or unmethylated based on the cut-off value determined using Youden’s J index (value combining highest sensitivity and specificity), through ROC curve analysis. Correlations between methylation levels and age were assessed by a Spearman nonparametric correlation test. A *p* value < 0.05 was considered statistically significant.

## Figures and Tables

**Table 1:** *RARβ2* index of methylation in the cirDNA from HFs and BCPs, mean ± SD *.

Blood Fraction	cirDNA	HFs (*n* = 10)	BCP (*n* = 8)	*p*
Plasma	100–1000 bp	ND	ND	
	Total	10 ± 3	33 ± 11	<0.05
PBS-EDTA eluate	100–1000 bp	ND	ND	
	Total	9 ± 4	15 ± 6	
Trypsin eluate	100–1000 bp	ND	ND	
	Total	7 ± 4	61 ± 12	<0.05

* ND—not detected.

**Table 2:** ROC area, cut-off value, specificity, and sensitivity for aberrantly methylated RARβ2 gene in cfDNA and blood csbDNA.

Sample	Methylated *RARβ2*, pg/mL	Sensitivity	Specificity	ROC-Area
cfDNA	70	52%	65%	0.711
csbDNA	330	74%	63%	0.752
Total blood DNA (cfDNA + csbDNA)	414	70%	61%	0.753

**Table 3:** Clinical characteristics of untreated BCPs.

		N (%)
Tumor stage	T1	8 (31%)
	T2	18 (69%)
Lymph node status	N0	19 (73%)
	N1	7 (21%)
Distant metastasis	M0	25 (96%)
	M1	1 (4%)
Molecular subtypes	Luminal A	13 (50%)
	Luminal B	13 (50%)
Histological type	Invasive ductal carcinoma	26 (100%)

**Table 4:** Sequences of primers used in Q-PCR.

Gene	Sequence (5′-3′)	
LINE1	Forward	TTCAACAAGAAGAGCTAACTATCC
	Reverse	TTGTAGGTCACTCAGGACTTGC
	Probe	[5,6]-TAMRA-TGCACCCAATACAGGAGCACCCAGATTCA-BHQ2
*RARβ2* GenBank X56849.1 926–1116, 161 b.p., dependent from methylation	Forward	AGG ATTGGGATGTCGAGAACGC
	Reverse	CTCGACCAATCCAACCGAAACG
*RARβ2* GenBank X56849.1 931–1116, 186 bp, undependent from methylation	Forward	TTGTTTGAGGATTGGGATG
	Reverse	TACCAT TTTCCAAACTTACTC
*RARβ2* GenBank X56849.1, 924–1117, 194 b.p., wild type	Forward	ATGCGAGCTGTTTGAGGACT
	Reverse	TTACCATTTTCCAGGCTTGC
